# PKM2 interacts with and phosphorylates PHB2 to sustain mitochondrial quality control against septic cerebral-cardiac injury

**DOI:** 10.7150/ijms.92367

**Published:** 2024-01-21

**Authors:** Yuanchen Zhao, Yawen Pan, Mengyuan Chen, Ying Tan, Xing Chang, Haixia Li, Yinghao Zhi

**Affiliations:** 1 Neurology Department, Wenzhou Hospital of Integrated Traditional Chinese and Western Medicine.; 2 Wenzhou TCM Hospital of Zhejiang Chinese Medical University, Wenzhou, 325000, China.; 3 Department of Cardiology, Chinese PLA General Hospital, Beijing, 100853, China.; 4 Guang'anmen Hospital, China Academy of Chinese Medical Sciences, Beijing, 100053, China.; 5 Department of Rehabilitation Medicine, Wenzhou TCM Hospital of Zhejiang Chinese Medical University, Wenzhou, 325000, China.

**Keywords:** PKM2, PHB2, mitochondrial quality control, septic cerebrovascular damage

## Abstract

Sepsis induces profound disruptions in cellular homeostasis, particularly impacting mitochondrial function in cardiovascular and cerebrovascular systems. This study elucidates the regulatory role of the Pyruvate Kinase M2 (PKM2)- Prohibitin 2 (PHB2) axis in mitochondrial quality control during septic challenges and its protective effects against myocardial and cerebral injuries. Employing LPS-induced mouse models, we demonstrate a significant downregulation of PKM2 and PHB2 in both heart and brain tissues post-sepsis, with corresponding impairments in mitochondrial dynamics, including fission, fusion, and mitophagy. Overexpression of PKM2 and PHB2 not only restores mitochondrial function, as evidenced by normalized ATP production and membrane potential but also confers resistance to oxidative stress by mitigating reactive oxygen species generation. These cellular mechanisms translate into substantial in vivo benefits, with transgenic mice overexpressing PKM2 or PHB2 displaying remarkable resistance to sepsis-induced cardiomyocyte and neuronal apoptosis, and organ dysfunction. Our findings highlight the PKM2-PHB2 interaction as a novel therapeutic target for sepsis, providing a foundation for future research into mitochondrial-based interventions to treat this condition. The study's insights into the molecular underpinnings of sepsis-induced organ failure pave the way for potential clinical applications in the management of sepsis and related pathologies.

## Introduction

Pyruvate Kinase M2 (PKM2) has emerged as a key metabolic enzyme transcending its traditional role in glycolysis 1. In the realm of cardiovascular and cerebrovascular health, particularly under the duress of sepsis, PKM2 assumes a critical regulatory function 2, 3. It acts not merely as a facilitator of aerobic glycolysis but also as a modulator of mitochondrial homeostasis and cell survival 4, 5. Recent insights have unraveled the intriguing dual functionality of PKM2 in endothelial cells 6 and cardiomyocytes 7. Under septic conditions, characterized by heightened inflammatory response and oxidative stress, PKM2 is pivotal in orchestrating a cellular adaptive response. This adaptation is crucial in maintaining endothelial integrity and cardiac function, thereby mitigating the catastrophic repercussions of septic cerebrovascular and cardiovascular damage.

Prohibitin 2 (PHB2) has increasingly been recognized as a central player in maintaining mitochondrial integrity and function, especially under pathological conditions such as sepsis 8, 9. In the intricate environment of cardiovascular and cerebrovascular systems, PHB2 serves not only as a structural component of the inner mitochondrial membrane but also as a crucial mediator in signaling pathways that govern cell survival and apoptosis 10, 11. Under septic conditions, characterized by systemic inflammation and oxidative stress, the role of PHB2 becomes particularly pivotal. It is instrumental in maintaining the mitochondrial morphology, ensuring efficient ATP production, and regulating oxidative stress responses 12. In the endothelial cells lining the blood vessels and the cardiomyocytes of the heart, PHB2's function is indispensable for sustaining cellular health and function 13, 14. The role of PHB2 in regulating mitochondrial quality control mechanisms, such as mitophagy, further highlights its importance 15. By influencing the selective degradation of damaged mitochondria, PHB2 ensures cellular homeostasis, a vital aspect in sepsis where cellular debris and dysfunction can exacerbate injury. Thus, exploring the multifaceted roles of PHB2 in the context of septic cardiovascular and cerebrovascular damage opens new avenues for understanding the pathophysiology of sepsis. It also underscores the potential of targeting mitochondrial pathways as a therapeutic strategy to ameliorate or prevent sepsis-induced damage in these critical systems.

Mitochondria, often termed the powerhouses of the cell, play a pivotal role in cardiovascular and cerebrovascular health. Their functions extend beyond mere ATP production; they are critical in regulating cellular metabolism, calcium homeostasis, reactive oxygen species (ROS) generation, and apoptotic pathways. This multifaceted involvement becomes especially significant in the context of septic conditions, where mitochondrial integrity and function are severely challenged. In the heart and brain vasculature, mitochondria are at the frontline of responding to the heightened metabolic demands and oxidative stress characteristic of sepsis 16. Their ability to efficiently produce ATP is essential for the high-energy demands of cardiac and cerebral tissues. Furthermore, the regulation of ROS by mitochondria is a double-edged sword; while low levels are crucial for cellular signaling, excessive ROS production during sepsis can lead to oxidative damage and cell death 17, 18. Mitochondrial dynamics, encompassing fission, fusion, and mitophagy, are vital in maintaining cellular homeostasis and responding to stress 19, 20. Under septic conditions, these processes are often dysregulated, contributing to mitochondrial dysfunction 21, 22. Dysfunctional mitochondria can exacerbate endothelial cell permeability and inflammation, leading to vascular leakage and organ failure, a hallmark of severe sepsis 23. The interplay between mitochondrial dysfunction and inflammation is a critical aspect of septic injury in cardiovascular and cerebrovascular systems. Mitochondria can release damage-associated molecular patterns (DAMPs), which trigger inflammatory responses, further exacerbating tissue damage.

This study is designed to elucidate the potential regulatory role of the PKM2-PHB2 axis in mitochondrial quality control and its subsequent impact on attenuating myocardial and cerebral injuries induced by sepsis. By investigating this interaction, we aim to deepen the understanding of the molecular mechanisms underpinning septic damage in these vital organ systems and potentially unveil novel therapeutic targets.

## Methods

### Mice and models

In this study, we employed both standard and genetically modified mouse models, including PKM2 transgenic (PKM2^Tg^) and PHB2 transgenic (PHB2^Tg^) strains, procured from The Jackson Laboratory. To induce a septic state in these mice, we administered an intraperitoneal injection of lipopolysaccharide (LPS) at a concentration of 15 mg/kg, aligning with methodologies outlined in prior research 24.

### Echocardiography

For echocardiographic analysis, we sedated the animals using a mild concentration (1.5%) of isoflurane delivered via a face mask. The Vevo2100 imaging system was utilized to measure left ventricular dimensions (both end-diastolic and end-systolic) and heart rates. We calculated fractional shortening using the formula: FS = 100 × ((LVEDD - LVESD)/LVEDD). Mice demonstrating a fractional shortening of less than 40% post-cryoinjury were included in the study 25. An investigator, unaware of the group allocations, conducted all echocardiographic assessments 26.

### ELISA

We seeded cells at a density of 10,000 per well in 96-well plates and allowed them to proliferate overnight. Post a 20-hour co-culture period, cell viability was assessed using the MTT assay, adhering to the guidelines provided by Promega 27. Additionally, caspase-3 activity, indicative of apoptosis, was quantified using an ELISA kit from Biolegend 28.

### RNA isolation and quantitative reverse transcription-PCR

RNA isolation was conducted using the Monarch Total RNA Miniprep Kit. For cDNA synthesis, 1 μg of total RNA underwent reverse transcription using Superscript III (Invitrogen), targeting gene expression profiling 29. Additionally, microRNA levels were assessed using 50 ng of RNA with the TaqMan MicroRNA Reverse Transcription Kit (Applied Biosystems). The PCR amplification followed standard protocols 30. Primer design for qRT-PCR was facilitated by Primer Express software, and the 2^-ΔΔCt^ method was employed to evaluate relative expression changes in genes and microRNAs 31.

### Cell culture

HL-1 and N2a cell lines, sourced from ATCC, underwent several passages before experimental use. Standard culture conditions included Dulbecco's Modified Eagle Medium (DMEM) enriched with penicillin (100 μg/ml), streptomycin (100 μg/ml), and Fetal Bovine Serum (FBS, 10%) from Gibco 32. The cells were incubated at a controlled temperature of 37°C with a 5% CO2 atmosphere. For in vitro sepsis simulation, the cells were exposed to LPS at a concentration of 10mg/ml 33.

### Histological analysis

Cardiac and cerebral tissues were meticulously extracted and immediately rinsed with saline. Subsequently, they were fixed using 4% paraformaldehyde. The fixed tissues were then embedded in paraffin for thin sectioning. Sections, with a thickness of 3-4 µm, were processed for histological analysis 34. This included hematoxylin and eosin (HE) staining for general morphological assessment and Nissl staining specifically aimed at evaluating neural integrity.

### Immunoprecipitation and western blot analysis

To stabilize proteins for immunoprecipitation, cells were first treated with MG132 for six hours, inhibiting proteasomal degradation 35. For lysis, we used a non-denaturing buffer containing 20 mM Tris-HCl (pH 7.5), 150 mM NaCl, 10% glycerol, 1% Triton X-100, and a protease inhibitor cocktail. Following overnight incubation with selected primary antibodies, samples were bound to IgG magnetic beads for two hours at 4°C with gentle agitation. The beads were then resuspended in Laemmli buffer, briefly heated for denaturation, and subjected to separation. For Western blot analysis, we quantified protein bands via integrated density measurements using ImageJ software, aligning with standard protocols 36.

### Construction of adenovirus vectors for overexpression of PHB2 and PKM2

The coding DNA sequences for mouse or human PKM2 and PHB2 were amplified via PCR from their respective plasmids and subsequently cloned into an adenovirus-Luciferase vector to create Ad-PKM2 and Ad-PHB2 constructs 37. The transfection process in HEK293 cells involved these constructs along with additional necessary plasmids using PEI. Post 72 hours, cells were harvested and lysed, followed by an overnight incubation with Adenovirus Enhanced Concentration Reagent at 4°C. The adenovirus vectors were then quantified, prepared in HN buffer, and used for subsequent subcutaneous inoculation at a determined viral load.

### Mitochondrial membrane potential and mitochondrial ROS detection

Mitochondrial membrane potential was evaluated in vitro by staining cells with tetramethylrhodamine methyl ester (TMRM, 200 nM) for 10 minutes. Post-staining, cells were imaged using a confocal microscope (Olympus FluoView 1000, UHN AOMF facility). Analysis was conducted using Image J, with results reflecting observations across multiple high-power fields from several biological replicates. For mitochondrial ROS detection, we employed a specific assay kit (CAYMAN CHEMICAL), ensuring robust and accurate quantification.

### Immunofluorescence staining

Cell samples were prepared according to a standard procedure and then incubated with blocking buffer containing 10% (w/v) bovine serum albumin (BSA) (NA8692, Bomei Biotechnology, Hefei, China). Afterward, the sections were incubated with primary antibodies overnight at 4°C and then with a fluorophore-conjugated secondary antibody (Alexa Fluor® 568 goat anti-rabbit IgG (H+L), Invitrogen A11036, Massachusetts, USA) for 1 h. Nuclei were stained with DAPI (0100-20, SouthernBiotech). Images were captured with a fluorescence microscope (BX51, Olympus, Japan). TUNEL staining was performed using a TUNEL Assay Kit for In Situ Apoptosis Detection (NO. C10617, Thermo Scientific).

### Statistics

Statistical evaluations were conducted using GraphPad Prism (Version 9). Data are presented as means ± standard deviation, except where otherwise specified. The sample size was not predetermined by statistical methods. Statistical significance was established at P values below 0.05.

## Results

### Suppression of PKM2 and PHB2 in Cardiac and Cerebral Tissues Post-Sepsis

**Figure 1:**
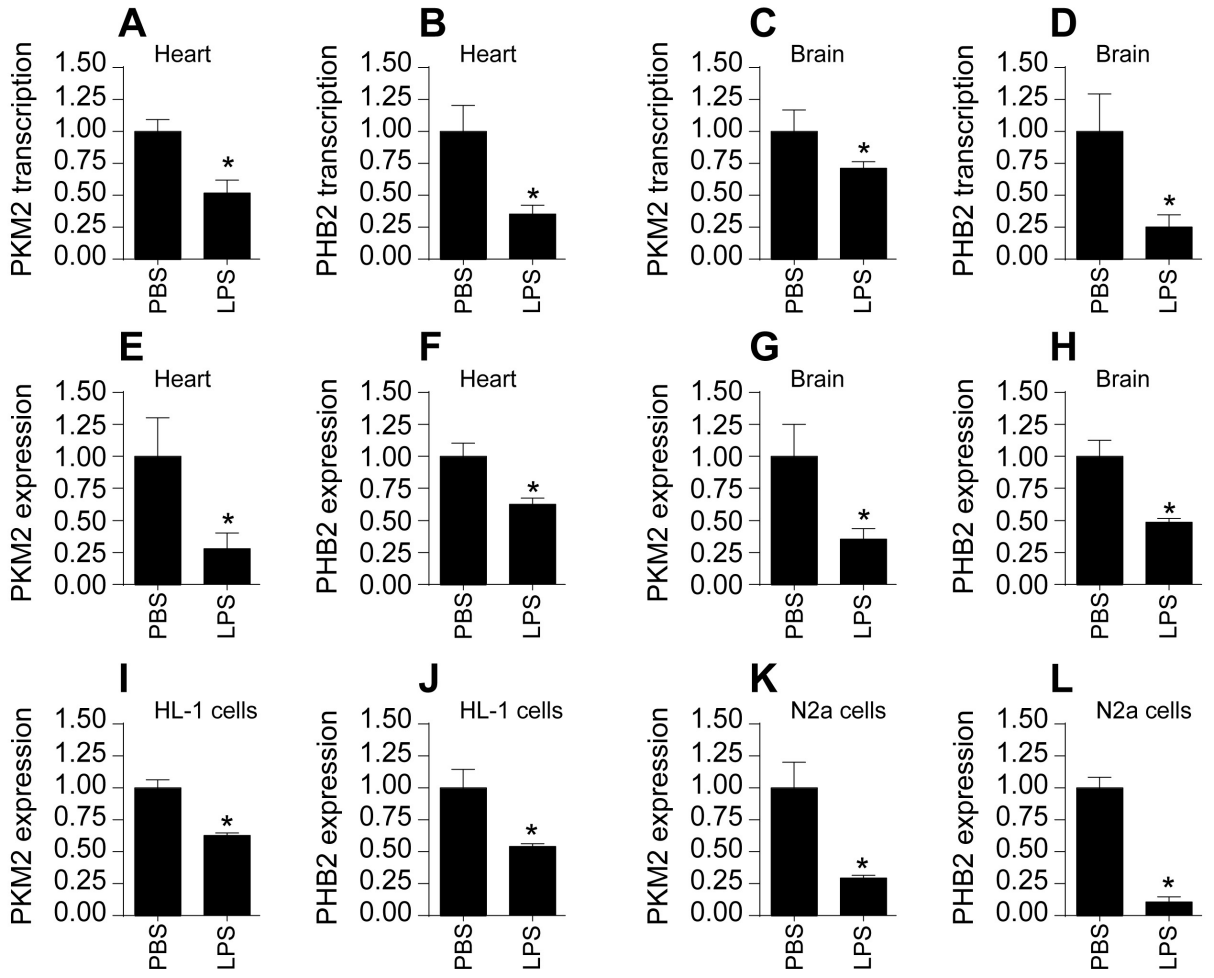
** Suppression of PKM2 and PHB2 in Cardiac and Cerebral Tissues Post-Sepsis. A-B.** RNA was isolated from heart tissues and then the transcriptions of PKM2 or PHB2 were measured via qPCR. **C-D.** RNA was isolated from brain tissues and then the transcription of PKM2 or PHB2 was measured via qPCR. **E-F.** Proteins were isolated from heart tissues and then the expression of PKM2 and PHB2 were determined by western blots.** G-H.** Proteins were isolated from brain tissues and then the expression of PKM2 and PHB2 were determined by western blots. **I-J.** Immunofluorescence of PKM2 or PHB2 in HL-1 cells. DAPI was used to stain nucleus. **K-L.** Immunofluorescence of PKM2 or PHB2 in HL-1 cells. DAPI was used to stain nucleus. *p<0.05 vs. PBS group.

To investigate alterations in PKM2 and PHB2 expressions in cardiac and cerebral tissues following sepsis, we utilized a lipopolysaccharide (LPS)-induced mouse model. Quantitative PCR (qPCR) analyses revealed a marked downregulation in the transcription levels of both PKM2 and PHB2 in these tissues upon septic challenge (Figure 1A-D). This transcriptional suppression was mirrored at the protein level, as evidenced by Western blot analyses. Notably, LPS-treated mice exhibited a significant reduction in PKM2 and PHB2 protein expressions compared to control mice receiving PBS (Figure 1E-H).

Further corroboration came from immunofluorescence studies. In cardiomyocytes from control mice, PKM2 and PHB2 were abundantly expressed; however, LPS treatment led to a notable suppression in the expression of these proteins (Figure 1I-J). This pattern was replicated in N2a neuroblastoma cells subjected to LPS treatment, with a marked decrease in PKM2 and PHB2 protein levels relative to baseline conditions (Figure 1K-L).

Collectively, these findings unequivocally demonstrate that sepsis, induced via LPS, leads to a significant downregulation of PKM2 and PHB2 in both heart and brain tissues.

### PKM2-PHB2 Interaction Preserves Mitochondrial Function during Inflammatory Stress

**Figure 2:**
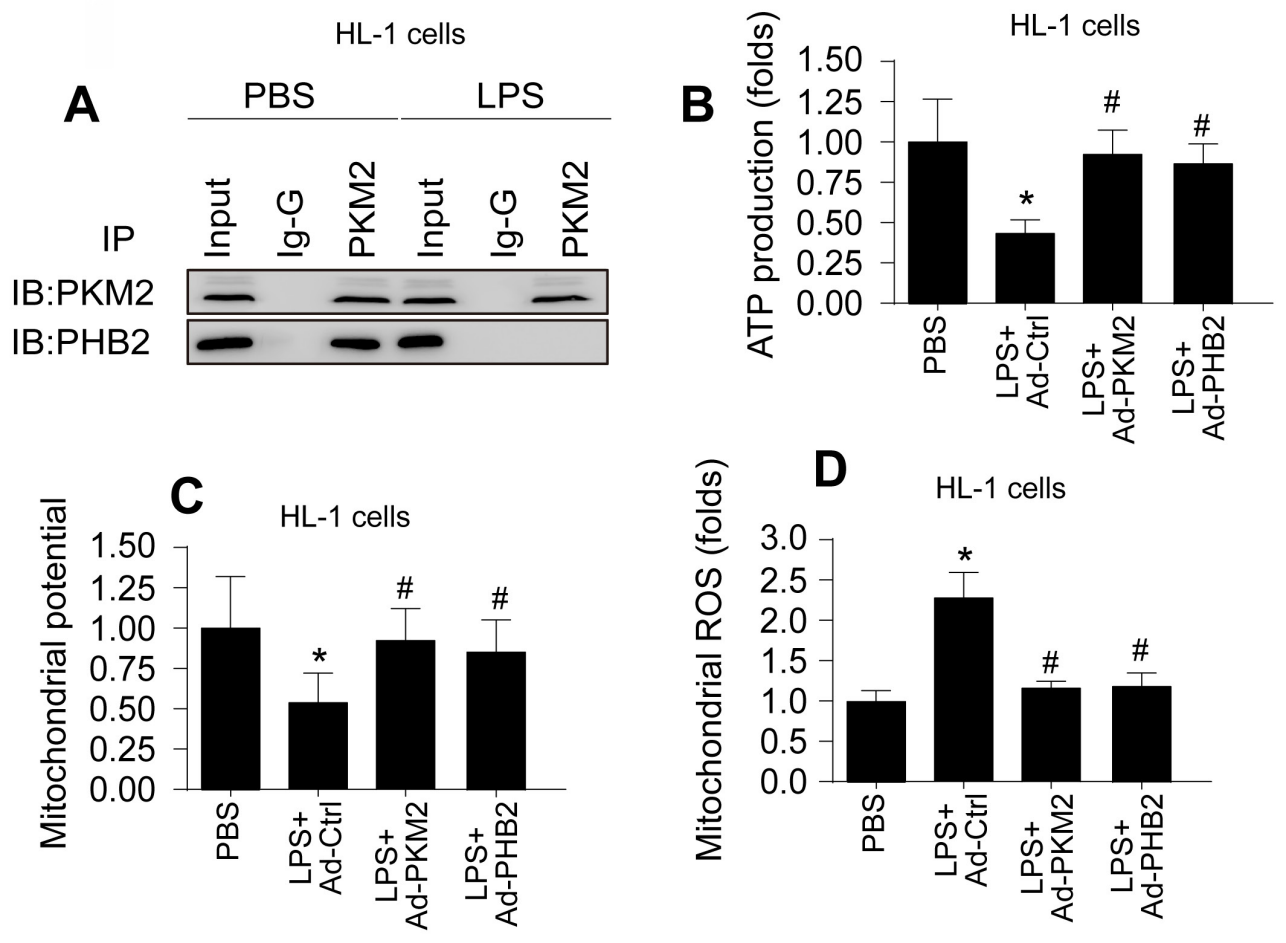
** PKM2-PHB2 Interaction Preserves Mitochondrial Function during Inflammatory Stress in HL-1 cells. A.** Co-IP assay was used to measure the interaction between PKM2 and PHB2 in HL-1 cells. **B.** ELISA kit was used to measure the ATP production in HL-1 cells.** C.** Immunofluorescence of mitochondrial membrane potential in HL-1 cells upon LPS exposure. **D.** Immunofluorescence of mROS in HL-1 cells upon LPS exposure. *p<0.05 vs. PBS group, #p<0.05 vs. LPS+Ad-Ctrl group.

**Figure 3:**
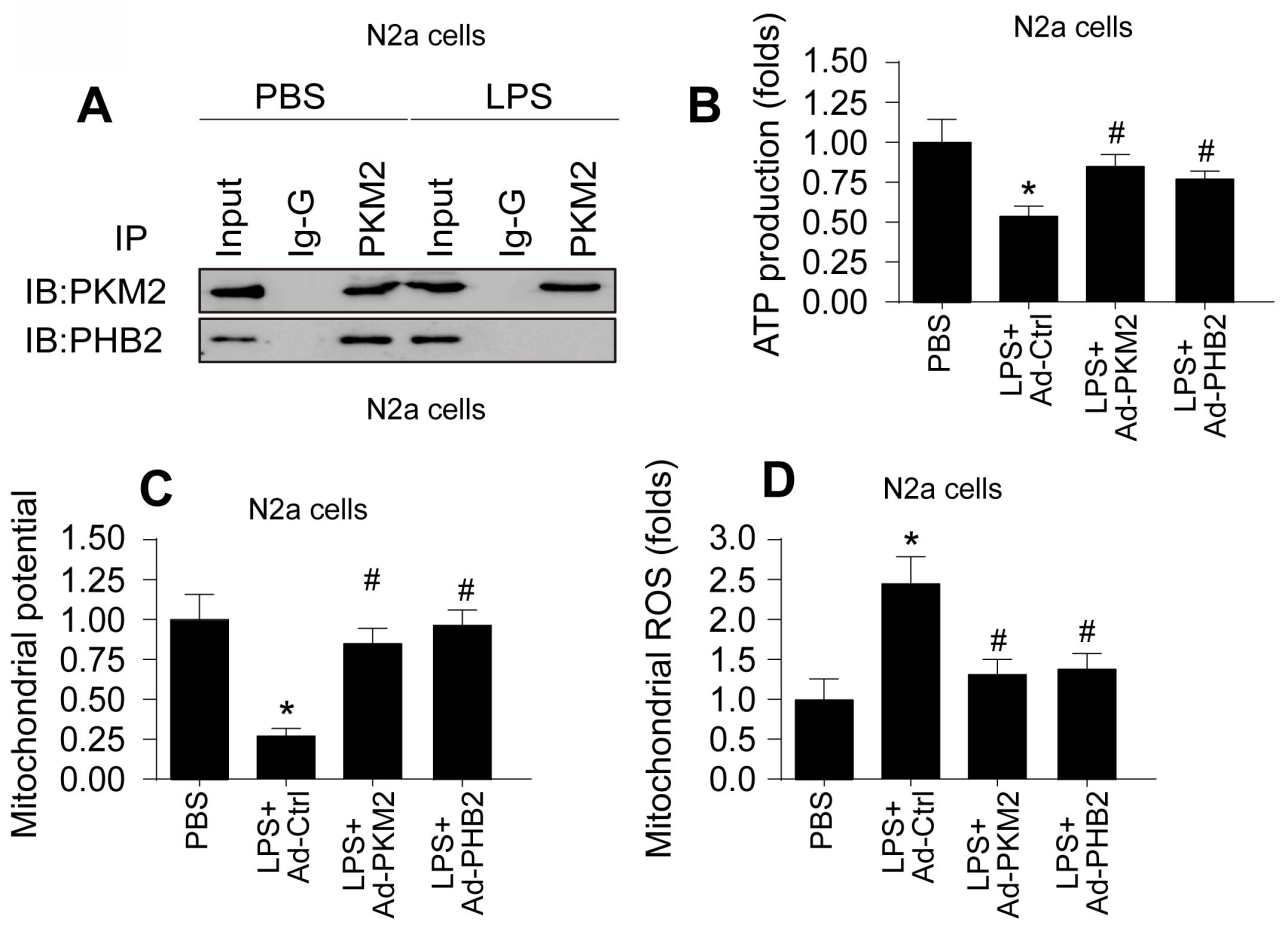
** PKM2-PHB2 Interaction Preserves Mitochondrial Function during Inflammatory Stress in N2a cells. A.** Co-IP assay was used to measure the interaction between PKM2 and PHB2 in N2a cells. **B.** ELISA kit was used to measure the ATP production in N2a cells.** C.** Immunofluorescence of mitochondrial membrane potential in N2a cells upon LPS exposure. **D.** Immunofluorescence of mROS in N2a cells upon LPS exposure. *p<0.05 vs. PBS group, #p<0.05 vs. LPS+Ad-Ctrl group.

Building upon previous research highlighting the roles of PKM2 and PHB2 in mitochondrial safeguarding, we explored the hypothesis that PKM2 interacts with PHB2 to protect against mitochondrial damage during LPS-induced inflammation. In HL-1 cardiomyocytes, co-immunoprecipitation (co-IP) assays confirmed this interaction, revealing that LPS disrupts the PKM2-PHB2 association (Figure 2A). A similar disruption in interaction was observed in N2a neuroblastoma cells under LPS treatment (Figure 3A).

To delineate the roles of PKM2 and PHB2 in mitochondrial preservation, we transfected HL-1 and N2a cells with adenoviruses carrying PKM2 or PHB2. Post-transfection, mitochondrial function was assessed via ATP synthesis measurement. Data indicated that LPS exposure significantly inhibited ATP production in both cell types (Figure 2B and Figure 3B). Notably, overexpression of PKM2 or PHB2 countered this effect, maintaining ATP levels.

Further investigations revealed that LPS treatment resulted in diminished mitochondrial membrane potential in both HL-1 (Figure 2C) and N2a cells (Figure 3C). However, cells overexpressing PKM2 or PHB2 exhibited stabilized mitochondrial membrane potentials (Figure 2C and Figure 3C).

Additionally, mitochondrial reactive oxygen species (ROS) production, a key indicator of mitochondrial oxidative stress, was markedly increased by LPS in both HL-1 cardiomyocytes (Figure 2D) and N2a cells (Figure 3D). Contrastingly, cells transfected with Ad-PKM2 or Ad-PHB2 showed resistance to LPS-induced ROS generation.

Collectively, these results underscore the protective role of the PKM2-PHB2 interaction in maintaining mitochondrial function under inflammatory stress induced by LPS.

### Association Between Reduced PKM2 and PHB2 Expression and Mitochondrial Quality Control Dysfunction in Sepsis-Induced Myocardial and Cerebral Injuries

**Figure 4:**
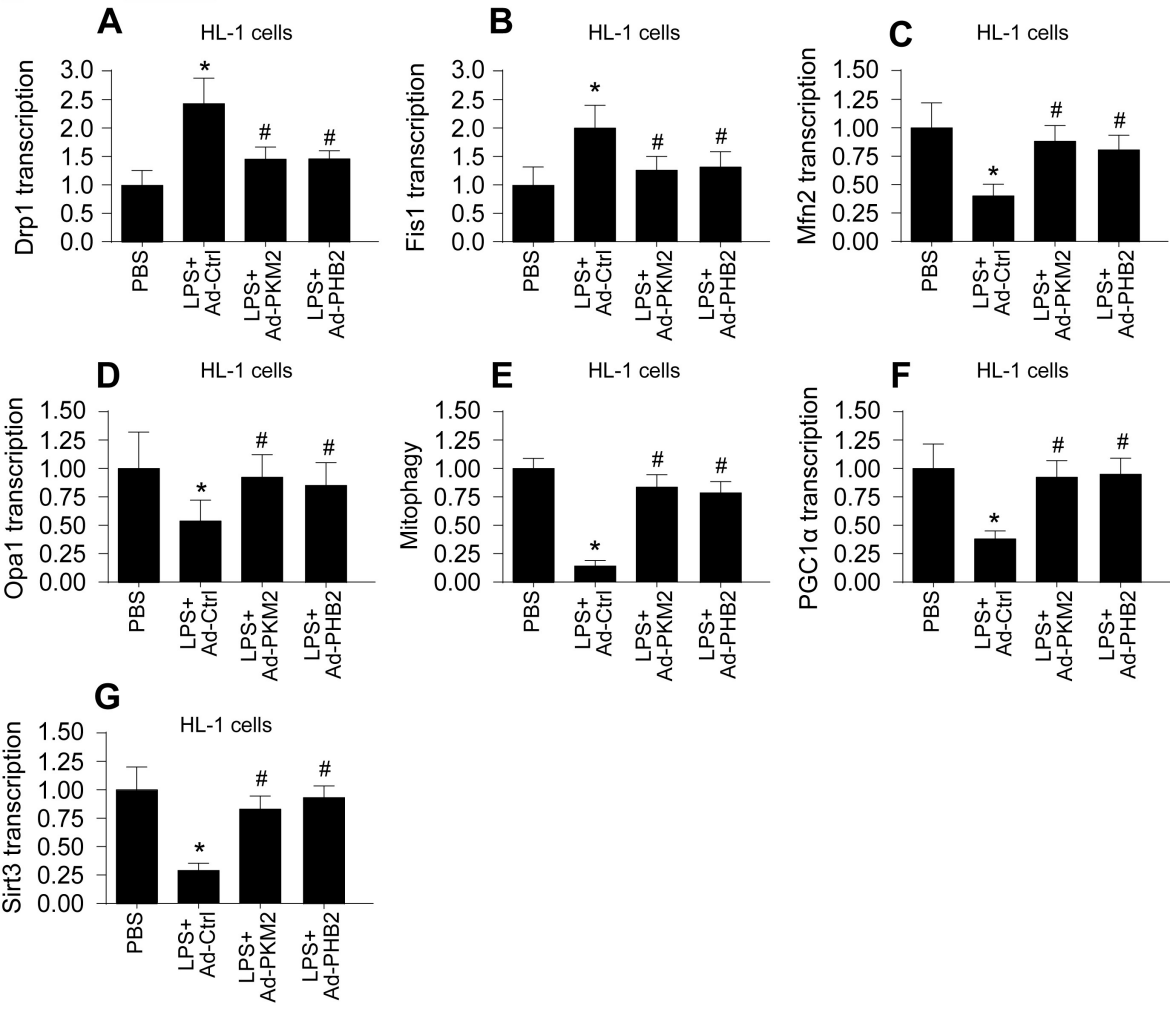
** Association Between Reduced PKM2 and PHB2 Expression and Mitochondrial Quality Control Dysfunction in LPS-treated HL-1 cells. A-D.** RNA was isolated from heart tissues and then the transcriptions of Drp1, Fis1, Mfn2 and Opa1 were measured via qPCR in LPS-treated HL-1 cells. **E.** The mitophagy activity was determined by mito-Kemia assay in LPS-treated HL-1 cells. **F-G.** RNA was isolated from heart tissues and then the transcriptions of Sirt3 and PGC1α were measured via qPCR in LPS-treated HL-1 cells. *p<0.05 vs. PBS group, #p<0.05 vs. LPS+Ad-Ctrl group.

**Figure 5:**
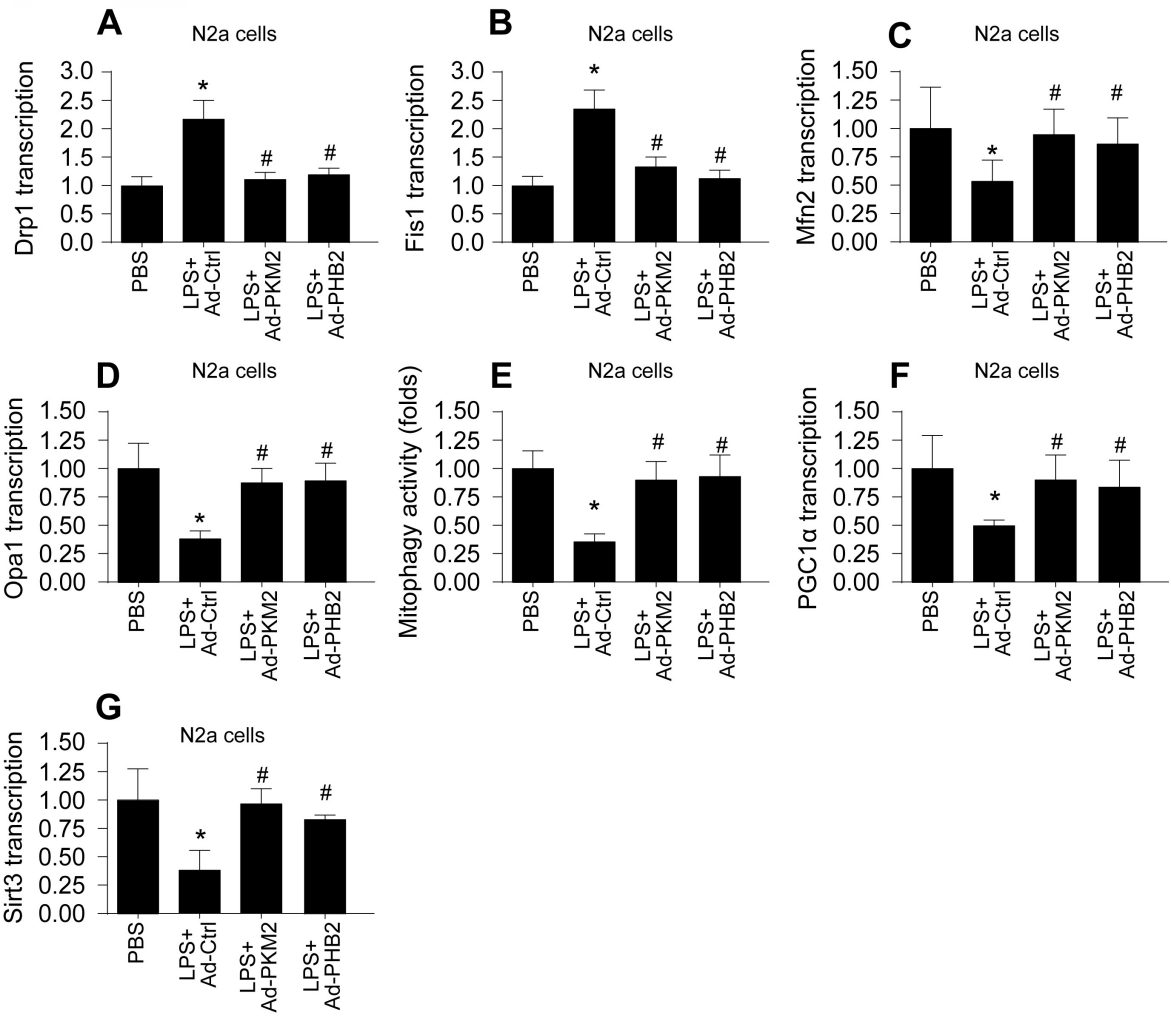
** Association Between Reduced PKM2 and PHB2 Expression and Mitochondrial Quality Control Dysfunction in LPS-treated N2a cells. A-D.** RNA was isolated from heart tissues and then the transcriptions of Drp1, Fis1, Mfn2 and Opa1 were measured via qPCR in LPS-treated N2a cells. **E.** The mitophagy activity was determined by mito-Kemia assay in LPS-treated N2a cells.** F-G.** RNA was isolated from heart tissues and then the transcriptions of Sirt3 and PGC1α were measured via qPCR in LPS-treated N2a cells. *p<0.05 vs. PBS group, #p<0.05 vs. LPS+Ad-Ctrl group.

Mitochondrial dysfunction, often resulting from impaired mitochondrial quality control, is a hallmark of sepsis-induced myocardial and cerebral injuries. We investigated whether PKM2 and PHB2 are integral to maintaining mitochondrial quality control under septic conditions. Initially, we assessed mitochondrial fission and fusion dynamics in HL-1 cardiomyocytes (Figure 4A-D) and N2a neuroblastoma cells (Figure 5A-D) using qPCR. LPS treatment led to an upregulation of fission markers Drp1 and Fis1 and a concomitant downregulation of fusion markers Mfn2 and Opa1 (Figure 4A-D and Figure 5A-D). Notably, adenovirus-mediated overexpression of PKM2 or PHB2 reversed these LPS-induced alterations in mitochondrial dynamics (Figure 4A-D and Figure 5A-D).

Furthermore, mitophagy was evaluated through mito-Kemia assay. LPS disrupted the interaction between mitochondria and lysosomes in both cell types, an effect that was mitigated by overexpressing PKM2 or PHB2 (Figure 4E and Figure 5E). This suggests that PKM2 and PHB2 are critical for maintaining mitophagy under inflammatory stress.

Lastly, we quantified mitochondrial biogenesis by measuring the transcription levels of Sirt3 and PGC1α. LPS exposure significantly reduced the expression of these biogenesis markers in both cell types (Figure 4F-G and Figure 5F-G). However, overexpression of PKM2 or PHB2 preserved the mRNA levels of these crucial biogenic factors (Figure 4F-G and Figure 5F-G).

In summary, our findings illuminate that a deficiency in PKM2 and PHB2 contributes to the dysregulation of mitochondrial quality control during LPS-induced sepsis, implicating these proteins as key modulators in septic myocardial and cerebral injuries.

### Overexpression of PKM2 and PHB2 Mitigates Sepsis-Induced Cardiomyocyte and Neuronal Death

**Figure 6:**
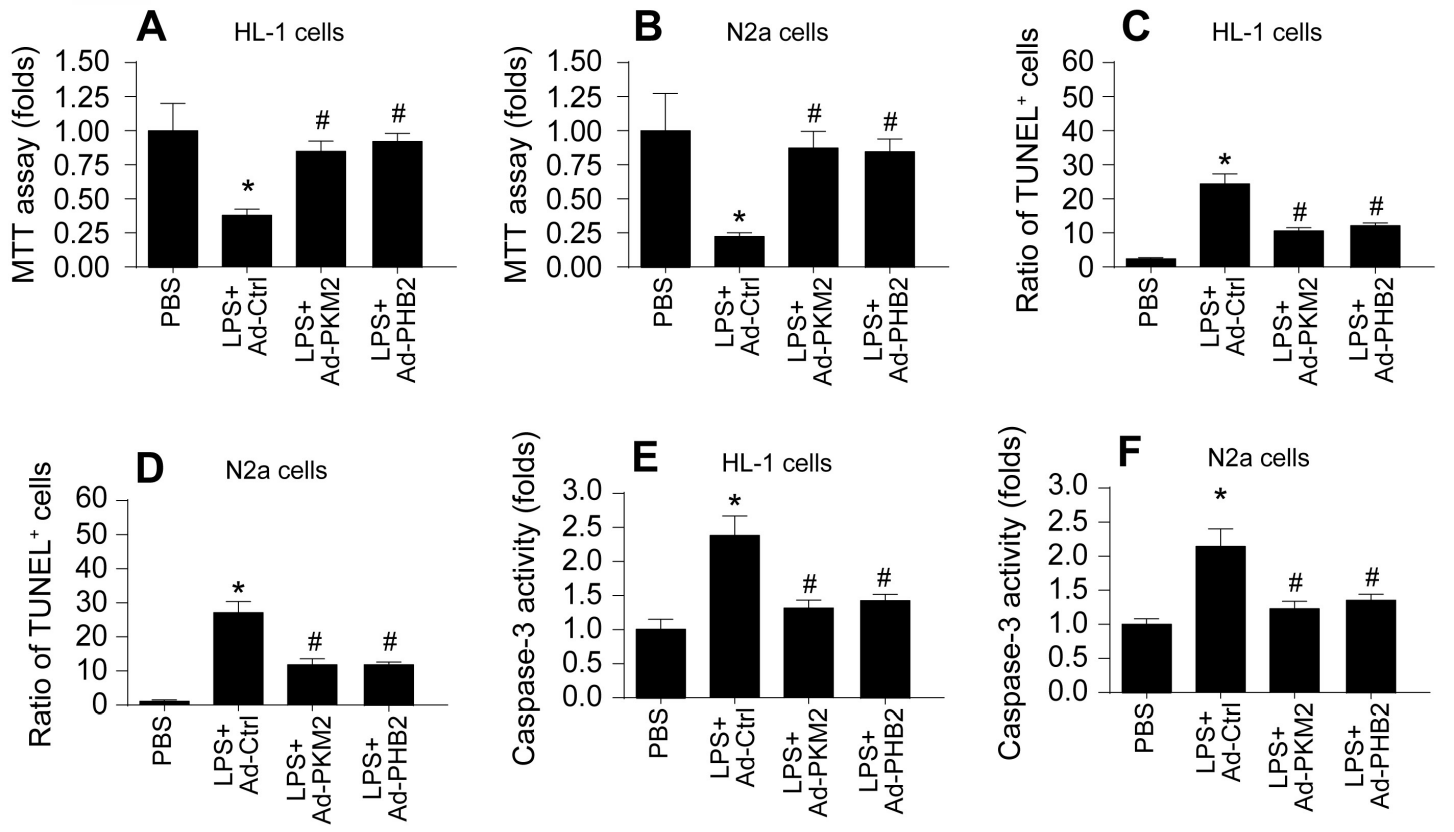
** Overexpression of PKM2 and PHB2 Mitigates Sepsis-Induced Cardiomyocyte and Neuronal Death. A-B.** Cell viability was determined by MTT assay in LPS-treated HL-1 cells or N2a cells. **C-F.** TUNEL staining of apoptotic cells in LPS-treated HL-1 cells or N2a cells. G-H. ELISA kit was used to detect the activity of caspase-3 in LPS-treated HL-1 cells or N2a cells. *p<0.05 vs. PBS group, #p<0.05 vs. LPS+Ad-Ctrl group.

This segment of our study aimed to discern if the mitochondrial protective effects conferred by PKM2 and PHB2 extend to preserving the viability and functional integrity of cardiomyocytes and neurons during septic conditions. We employed a series of in vitro assays to assess cell viability and apoptosis in HL-1 cardiomyocytes and N2a neuroblastoma cells. Cell viability assays, using the Cell Counting Kit-8 (CCK-8), revealed a substantial decline in the viability of both HL-1 and N2a cells following LPS treatment (Figure 6A-B). Contrastingly, transfection with adenoviruses encoding PKM2 (Ad-PKM2) or PHB2 (Ad-PHB2) significantly preserved cell viability in these models.

Further, Terminal deoxynucleotidyl transferase dUTP nick end labeling (TUNEL) staining indicated an increase in apoptosis rates in cardiomyocytes post-LPS exposure, a trend that was notably reversed upon transfection with Ad-PKM2 or Ad-PHB2 (Figure 6C-D). A similar pattern was observed in N2a cells, where LPS-induced apoptosis was mitigated by the overexpression of PKM2 or PHB2. Moreover, enzyme-linked immunosorbent assay (ELISA) results showed that caspase-3 activity, a critical mediator of apoptosis, was significantly elevated in both HL-1 and N2a cells after LPS treatment (Figure 6E-F). However, transfection with Ad-PKM2 or Ad-PHB2 effectively inhibited this caspase-3 activation (Figure 6E-F).

These findings collectively substantiate the hypothesis that overexpression of PKM2 and PHB2 plays a crucial role in averting sepsis-induced cardiomyocyte and neuronal apoptosis, thereby preserving cellular integrity and function in the face of septic challenges.

### PKM2 Overexpression Ameliorates Sepsis-Induced Cardiovascular and Cerebral Dysfunction In Vivo

**Figure 7:**
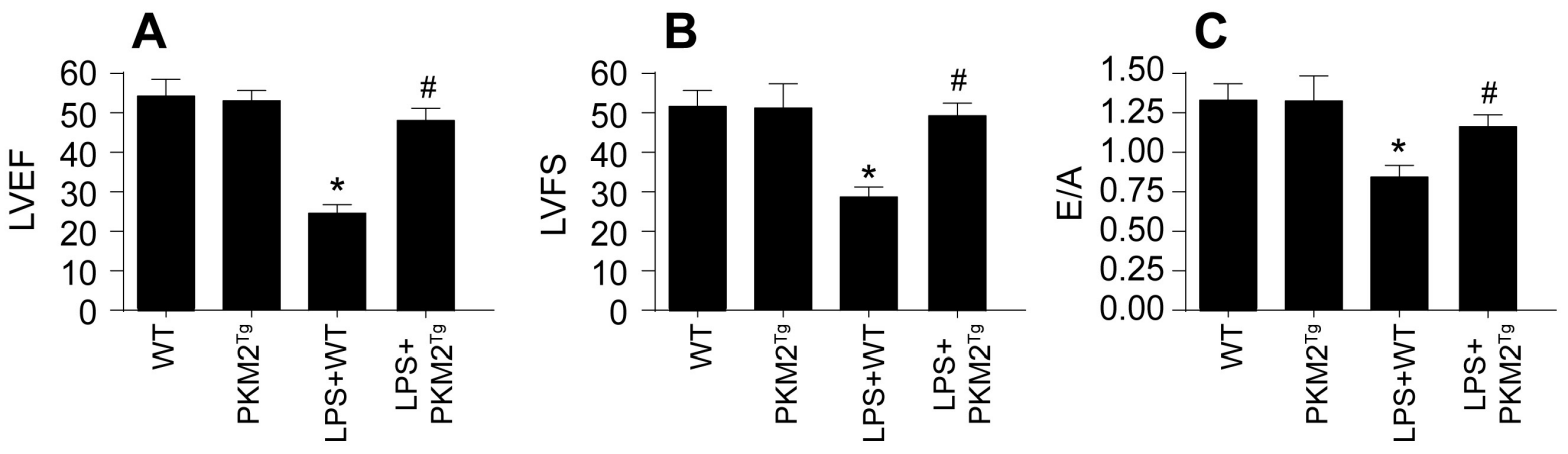
** PKM2 Overexpression Ameliorates Sepsis-Induced Cardiovascular and Cerebral Dysfunction In Vivo. A-C.** Heart function was measured via echocardiography. *p<0.05 vs. WT group, #p<0.05 vs. LPS+WT group.

In an effort to translate our in vitro findings into an in vivo context, we utilized PKM2 transgenic (PKM2^Tg^) mice subjected to LPS-induced sepsis. Cardiac function in these mice was evaluated using echocardiography. Notably, LPS treatment in wild-type (WT) mice led to a marked deterioration in cardiac performance (Figure 7A-C), as indicated by reduced left ventricular ejection fraction (LVEF) and left ventricular fractional shortening (LVFS), and impaired diastolic function, assessed by the E/A ratio (Figure 7A-C). Conversely, PKM2Tg mice exhibited resilience to LPS-induced cardiac dysfunction. Collectively, these in vivo results substantiate the protective role of PKM2 overexpression in countering the adverse effects of sepsis on myocardial and cerebral functions, highlighting its potential therapeutic value in managing sepsis-induced organ dysfunction.

### PHB2 Overexpression Confers Resilience Against Sepsis-Induced Cardiovascular and Cerebral Dysfunction

**Figure 8:**
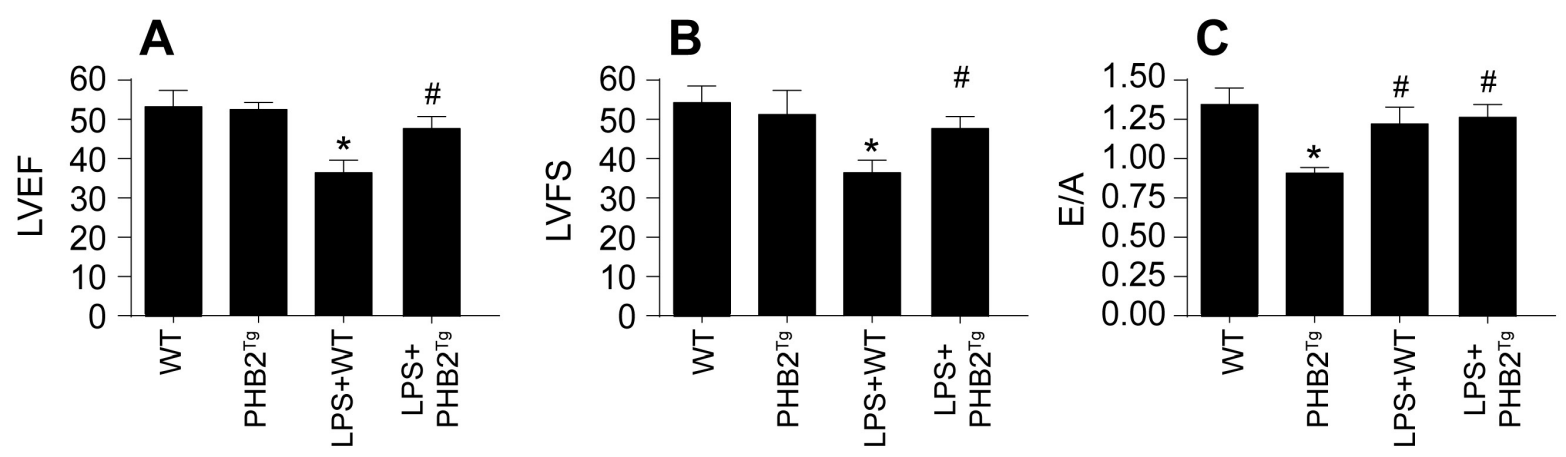
** PHB2 Overexpression Confers Resilience Against Sepsis-Induced Cardiovascular and Cerebral Dysfunction. A-C.** Heart function was measured via echocardiography. *p<0.05 vs. WT group, #p<0.05 vs. LPS+WT group.

In a bid to extend our in vitro observations to an in vivo model, we engineered PHB2 transgenic (PHB2^Tg^) mice and subjected them to LPS-induced sepsis. Cardiac function was assessed via echocardiography. It was found that LPS administration significantly compromised cardiac function in wild-type (WT) mice, as manifested by a decrease in left ventricular ejection fraction (LVEF) and left ventricular fractional shortening (LVFS), alongside a diminished heart relaxation capacity, quantified by the E/A ratio (Figure 8A-C). Remarkably, such cardiac impairments were not observed in PHB2^Tg^ mice. Overall, our findings provide compelling evidence that PHB2 overexpression mitigates the detrimental effects of sepsis on myocardial and cerebral functions, suggesting a potential therapeutic strategy for sepsis-induced organ dysfunction.

## Discussion

The present investigation delves into the molecular interplay between PKM2 and PHB2, providing novel insights into their roles in mitochondrial quality control and the pathophysiology of septic cerebrovascular damage. Our findings demonstrate that sepsis induces a profound suppression of PKM2 and PHB2, corroborating their roles as mitochondrial protectants and signaling entities. Notably, the overexpression of these proteins mitigates the septic insult, preserving myocardial and cerebral functions, which signifies a potential therapeutic pathway.

The observed downregulation of PKM2 and PHB2 at both transcriptional and protein levels post-sepsis suggests that these molecules may be sensitive indicators of septic stress in cardiac and cerebral tissues. The restoration of mitochondrial function via PKM2 and PHB2 overexpression, as evidenced by stabilized mitochondrial membrane potentials and normalized ATP production, underscores the critical role of the PKM2-PHB2 axis in cellular energy homeostasis.

It is particularly noteworthy that the PKM2-PHB2 interaction serves as a bulwark against LPS-induced mitochondrial fragmentation and dysfunction. This is evidenced by the reversal of sepsis-associated alterations in mitochondrial dynamics, such as the balance between fission and fusion, and the maintenance of mitophagy. These processes are essential for the removal of damaged mitochondria, thus preventing the release of pro-inflammatory mitochondrial DAMPs, which can exacerbate sepsis pathology 38, 39. Intriguingly, our* in vivo* findings reveal that PKM2 and PHB2 transgenic mice exhibit resilience to sepsis-induced cardiac and cerebral dysfunction. The absence of myocardial edema and neural degradation in these transgenic models upon LPS exposure suggests that PKM2 and PHB2 confer structural and functional protection against septic damage.

The therapeutic implications of these findings are profound. By enhancing the expression of PKM2 and PHB2, it may be possible to bolster mitochondrial quality control mechanisms, thus offering a novel strategy to counteract the mitochondrial and cellular dysfunctions inherent in sepsis 2, 3. This approach may not only safeguard organ integrity but also improve outcomes in patients suffering from septic shock.

The compelling evidence provided by this study on the protective roles of PKM2 and PHB2 in mitigating sepsis-induced mitochondrial dysregulation opens new avenues for clinical intervention strategies. The translational potential of these findings lies in their ability to inform the development of novel therapeutic agents that can preserve mitochondrial integrity and function during septic challenges 40, 41. The modulation of PKM2 and PHB2 presents a promising therapeutic target for the development of drugs aimed at enhancing mitochondrial resilience. Agents that can mimic or enhance the activity of these proteins could potentially mitigate the effects of sepsis-induced organ dysfunction. Given the downregulation of PKM2 and PHB2 during sepsis, these proteins could serve as biomarkers for early detection and the progression of septic injury. Their levels could be monitored to gauge the severity of the condition and to tailor individualized treatment plans 20, 42-45. The use of gene therapy to overexpress PKM2 and PHB2 in susceptible patient populations, such as those with a history of sepsis or at high risk of developing septic complications, could be explored. This would be particularly useful in preserving organ function and preventing the progression of sepsis. The differential expression of PKM2 and PHB2 in response to septic insult may pave the way for personalized medical approaches. Therapeutics could be tailored based on an individual's expression profile of these proteins, optimizing treatment efficacy and minimizing potential side effects. In conjunction with standard sepsis treatments, drugs targeting PKM2 and PHB2 could be part of a combination therapy approach. Such a strategy could enhance the overall treatment outcome by simultaneously addressing multiple pathways involved in sepsis pathophysiology. The transition from preclinical models to clinical trials will require a concerted effort to understand the dosing, timing, and delivery mechanisms of PKM2 and PHB2 modulators 46-49. This will necessitate rigorous clinical trials to establish safety and efficacy in humans. The principles derived from this study could extend beyond sepsis to other diseases where mitochondrial dysfunction is a key player, such as neurodegenerative diseases, myocardial infarction, and stroke. This broadens the impact of the current findings and underscores the universality of mitochondrial health in clinical outcomes 50-52.

However, the present study is not without limitations. The exact mechanistic pathways through which PKM2 and PHB2 exert their protective effects warrant further investigation. Moreover, while our transgenic models offer valuable insights, the translatability of these findings to the clinical setting requires additional exploration, particularly in the context of patient heterogeneity and the complexity of sepsis as a systemic inflammatory response. Future research should aim to elucidate the detailed molecular mechanisms underlying the PKM2-PHB2 interaction and its impact on mitochondrial dynamics. Furthermore, clinical trials are imperative to assess the efficacy of modulating PKM2 and PHB2 expressions as a therapeutic intervention for sepsis. As we advance our understanding, it becomes increasingly clear that targeting mitochondrial quality control could be a key to unlocking new treatments for this devastating condition.

In summary, the study not only sheds light on the fundamental aspects of mitochondrial quality control in sepsis but also provides a strong foundation for future research and development of novel clinical therapies. As we continue to unravel the complexities of sepsis and its impact on cellular metabolism, the horizon for clinical translation of these findings remains promising and warrants further exploration.

## Funding

This study is supported by the fifth batch of national Traditional Chinese Medicine clinical outstanding talents training project (National Administration of Traditional Chinese Medicine talent education letter No.2022), and the Zhejiang Province Traditional Chinese Medicine Inheritance and Innovation Team for Diagnosis and Treatment of Cerebrovascular Diseases.
